# Supplementary figures and images for: Endothelial cell signature in muscle stem cells validated by VEGFA-FLT1-AKT1 axis promoting survival of muscle stem cell
Source: eLife. 2024 Jun 6;13:e73592. doi: 10.7554/eLife.73592 (PMC11216748; doi:10.7554/eLife.73592)

Figure 2-figure supplement 1N source data

Uncropped blotting image

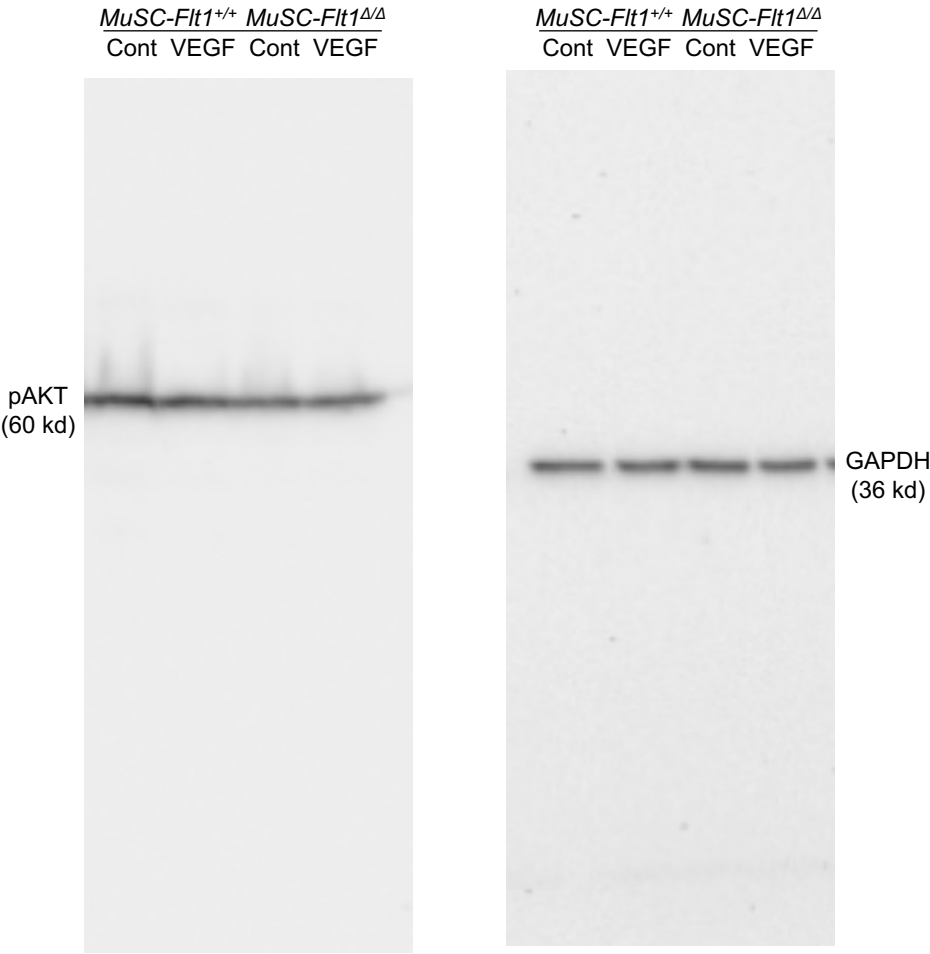

Supplement: Figure 2—figure supplement 1—source data 2. [file elife-73592-fig2-figsupp1-data2.pdf]
