# Supplementary material for: Endothelial cell signature in muscle stem cells validated by VEGFA-FLT1-AKT1 axis promoting survival of muscle stem cell
Source: eLife. 2024 Jun 6;13:e73592. doi: 10.7554/eLife.73592 (PMC11216748; doi:10.7554/eLife.73592)
Supplement: Supplementary file 1. [file elife-73592-supp1.docx]

**Supplementary Table 1**

| **Gene Expression Omnibus (GEO)** |  |  |
| --- | --- | --- |
| **Bulk RNAseq, scRNAseq or Microarrays** | **Repository** | **Original** |
| scRNAseq of MuSC and muscle ECs | GSE129057 | Current manuscript |
| scRNAseq of whole muscle | GSE143437 | De Micheli et al., 2020 |
| Bulk RNAseq of MuSCs, ECs and single muscle fibers | GSE108739 | Verma et al., 2018 |
| Bulk RNAseq of TU-tagged RNA of MuSCs | GSE97399 | van Velthoven et al., 2017 |
| Microarrays of human muscle diseases related to neuromuscular diseases | GSE3307 | Bakay et al., 2006; Dadgar et al., 2014 |
| Microarrays of human DMD muscle | GSE465 | Chen et al, 2000 |
| Microarrays of *mdx* muscle | GSE466 | Tseng et al., 2002 |
| Microarrays of DMD dog muscle | GSE69040 | Vieira et al., 2015; Vieira et al., 2017 |
| Microarrays of *mdx* MuSCs | GSE15155 | Pallafacchina et al., 2010; L'honoré et al., 2018 |
| Bulk RNAseq of fixed and unfixed MuSCs | GSE113631 | Yue et al., 2020 |
| Microarrays of MuSCs | GSE3483 | Fukada et al., 2007 |
| Bulk RNAseq of MuSCs | GSE64379 | Ryall et al., 2015 |
